# A Case Report and Literature Review of a Proliferating Pilar Tumor (PPT) of the Scalp

**DOI:** 10.7759/cureus.90621

**Published:** 2025-08-20

**Authors:** Bassem Y Sheikh, Areej A Aljohani, Maamoun M Khoja, Malik M Alrashedi

**Affiliations:** 1 Department of Neurosurgery, College of Medicine, Taibah University, Medina, SAU; 2 Department of Neurosurgery, King Salman bin Abdulaziz Medical City, Medina, SAU; 3 Department of Histopathology, King Salman bin Abdulaziz Medical City, Medina, SAU

**Keywords:** high-grade malignant ppt, locally malignant ptt, proliferating pilar tumors, proliferating trichilemmal tumor, scalp mass

## Abstract

Proliferating pilar tumor (PPT) or proliferating trichilemmal tumor is a rare type of cutaneous tumors that predominantly affect the scalp of elderly women with an autosomal dominant pattern. However, no standardized management guidelines have been developed yet. In this article, we reported a rare incidence of the disease among Saudi Arabia's population with a literature review. A 40-year-old female patient presented with multiple and recurrent scalp swellings that were diagnosed as benign PPT and treated with repeated surgical excision.

## Introduction

The proliferating pilar/trichilemmal tumors (PPTs/PTTs) are slowly growing masses of the outer root sheath of hair follicles [1]. PTTs are challenging to diagnose, as they share various similar features on clinical and histological bases with squamous cell carcinoma (SCC) and trichilemmal cysts (TCs) [1]. However, the absence of a granular layer with secondary abrupt trichilemmal keratinization is a hallmark of PTT and proliferating trichilemmal cyst. Further immunohistochemical staining is then required to confirm the diagnosis of PTT [1,2]. Other differentials include keratinous or sebaceous cysts, trichilemmal keratosis, clear-cell syringoma, hidradenoma, and other conditions [3]. Usually, it affects elderly women on a larger scale [3] and is frequently located in the scalp, with variable consistency ranging from solid to partially cystic, with the potential to ulcerate [4]. The tumor size varies from 2 to 15 cm, but a maximum size of 24 cm has been reported in the literature [4]. Single PTT is commonly sporadic; on the other hand, multiple PTTs tend to show genetic inheritance with some involved genes identified as PLCD1 [5]. We report a case of a 40-year-old female patient who presented with multiple and recurrent scattered scalp lesions that had been increasing in size gradually over four years. She had a previous history of a scalp cyst removed surgically 11 years ago, with a positive family history in several members. To our knowledge, this case is the first to be reported in the literature among the Saudi Arabian population.

## Case presentation

A 40-year-old female patient presents to the clinic with a complaint of chronic scalp lumps. The first single lump was discovered in the occipital area 11 years ago in 2014 and was surgically removed at the time with the first suspicion of simple pilar cysts. Nevertheless, histopathological analysis at that time was consistent with PTT. Then, over four years from 2020 to 2024, multiple scalp swellings had slowly grown again, but with multiple sizes and locations. Hence, during the follow-up visit, the patient complained the most about one lump, which was in the occipital area, that was interfering with sleep due to its large size and tenderness when compressed during sleep, which led her to seek this surgical excision. Upon physical examination, the cysts are variable in size and location, with the largest being at the left occipital region (28 x 18 mm) and the smallest (5 x 4 mm) in the frontal area. All lumps are confined to the hair-bearing scalp. All masses were soft and mobile, and not anchored to the underlying tissue; the large mass was tender. The overlying skin was reddish with some crust and small ulcers, as patients stated that some of the mass had "ruptured," secreting thick fluid and then decreased in size. No cervical lymph node enlargement was noted. No other similar cysts in other body parts were noticed.

She mentioned a positive family history as her father, sisters, and grandmother suffer from the same condition. However, no histopathological reports or confirmed diagnosis were available.

**Figure 1:**
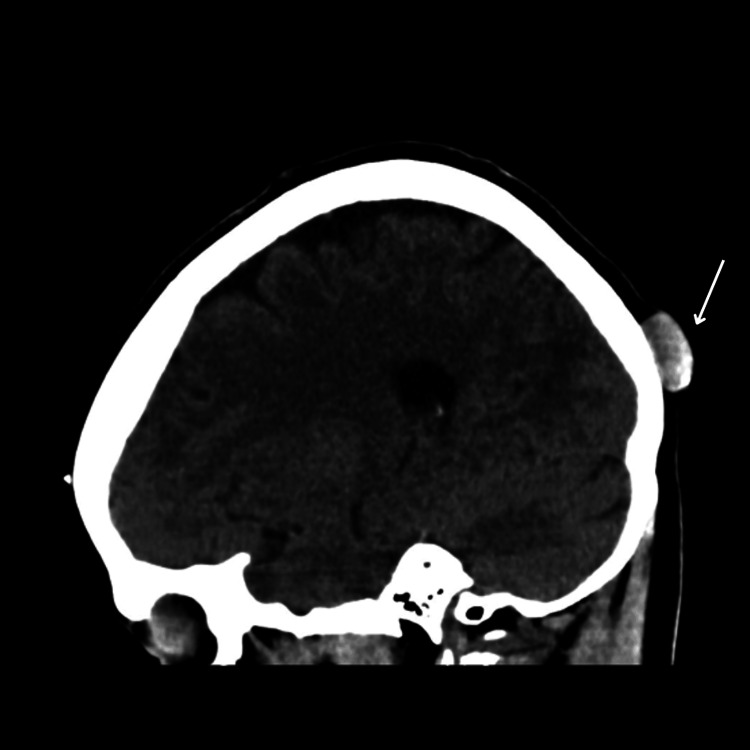
Sagittal view of the noncontrasted CT scan of head Left occipital well-demarcated round lesion with partial calcification without skull invasion (arrow) CT: computed tomography

**Figure 2:**
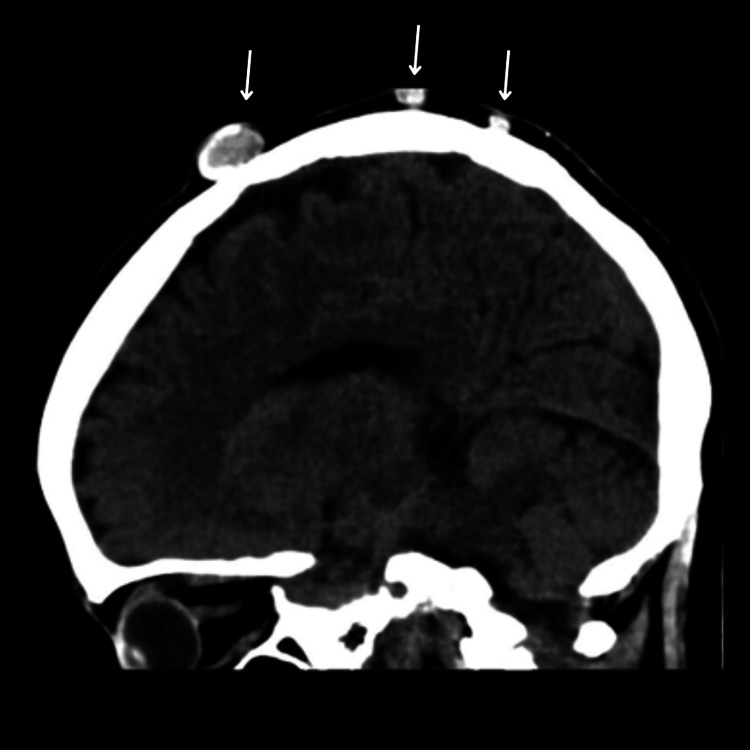
Sagittal view on the noncontrasted CT scan of head Three masses varying in size and depth at the top of the vertex, with partial calcification (arrows) CT: computed tomography

**Figure 3:**
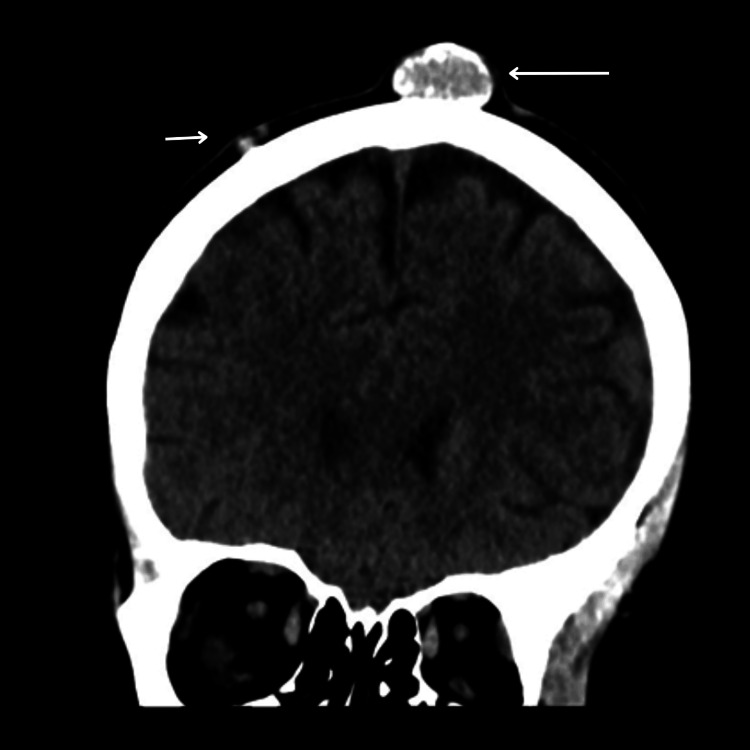
Coronal view of the noncontrasted CT scan of the head A large midline solid cyst with partial calcification is noted. Two small masses in the right parietal area are noted (arrows) CT: computed tomography

Contrast computed tomography (CT) scan of the head revealed 12 bilateral, well-defined, partially calcified cystic lesions of varying sizes scattered across the scalp, with no intracranial extension and intact underlying bone (Figures 1-3). The largest was seen at the left occipital region.

Based on patient history and previous resection, recurrent PTT was at the top of the differentials. The patient had a second surgical resection of large cysts, specifically the left occipital and right parietal lesions, to provide symptomatic relief and obtain further tissue diagnosis to confirm recurrence.

**Figure 4:**
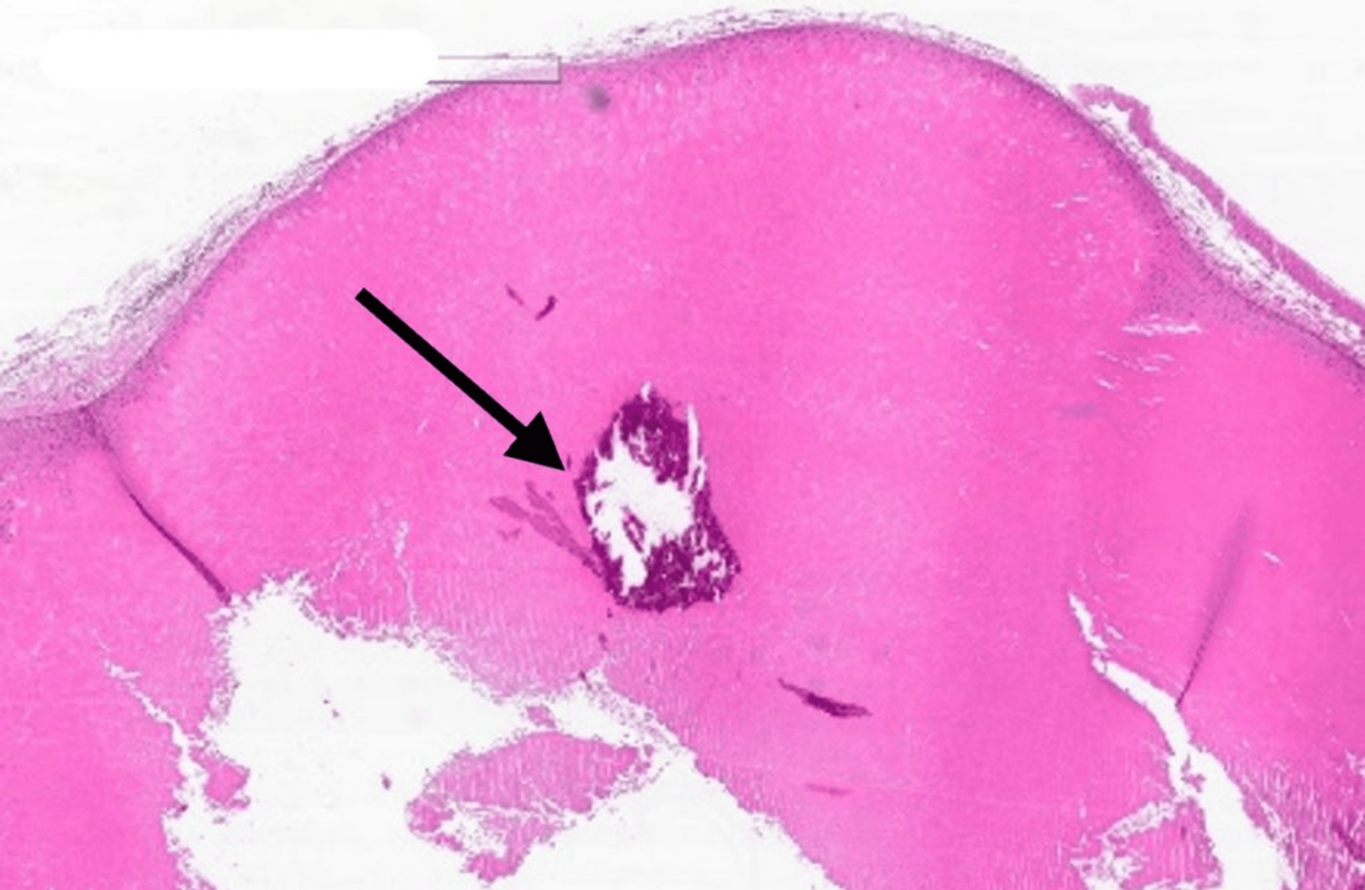
An intralesional cyst with surrounding calcification and hemorrhage (magnified ×4) (black arrow)

**Figure 5:**
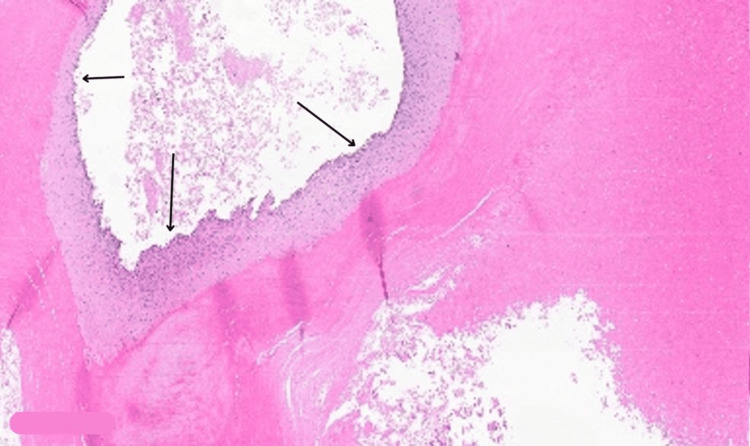
The lining epithelium (stratified squamous epithelium) exhibiting abrupt trichilemmal keratinization

**Figure 6:**
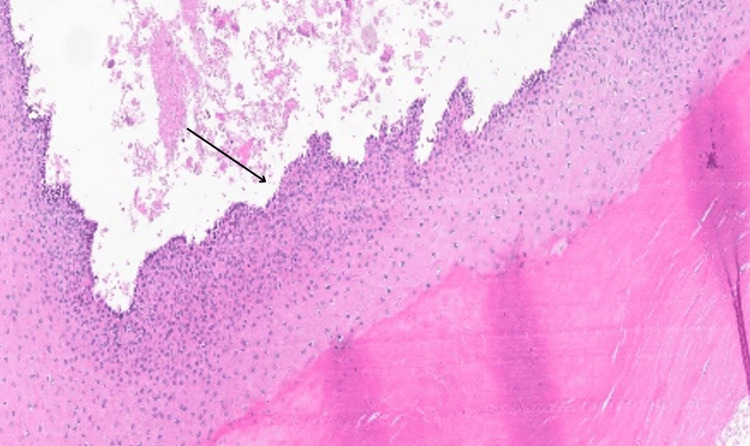
Lining epithelium of cystic island (magnified ×10) (black arrow)

**Figure 7:**
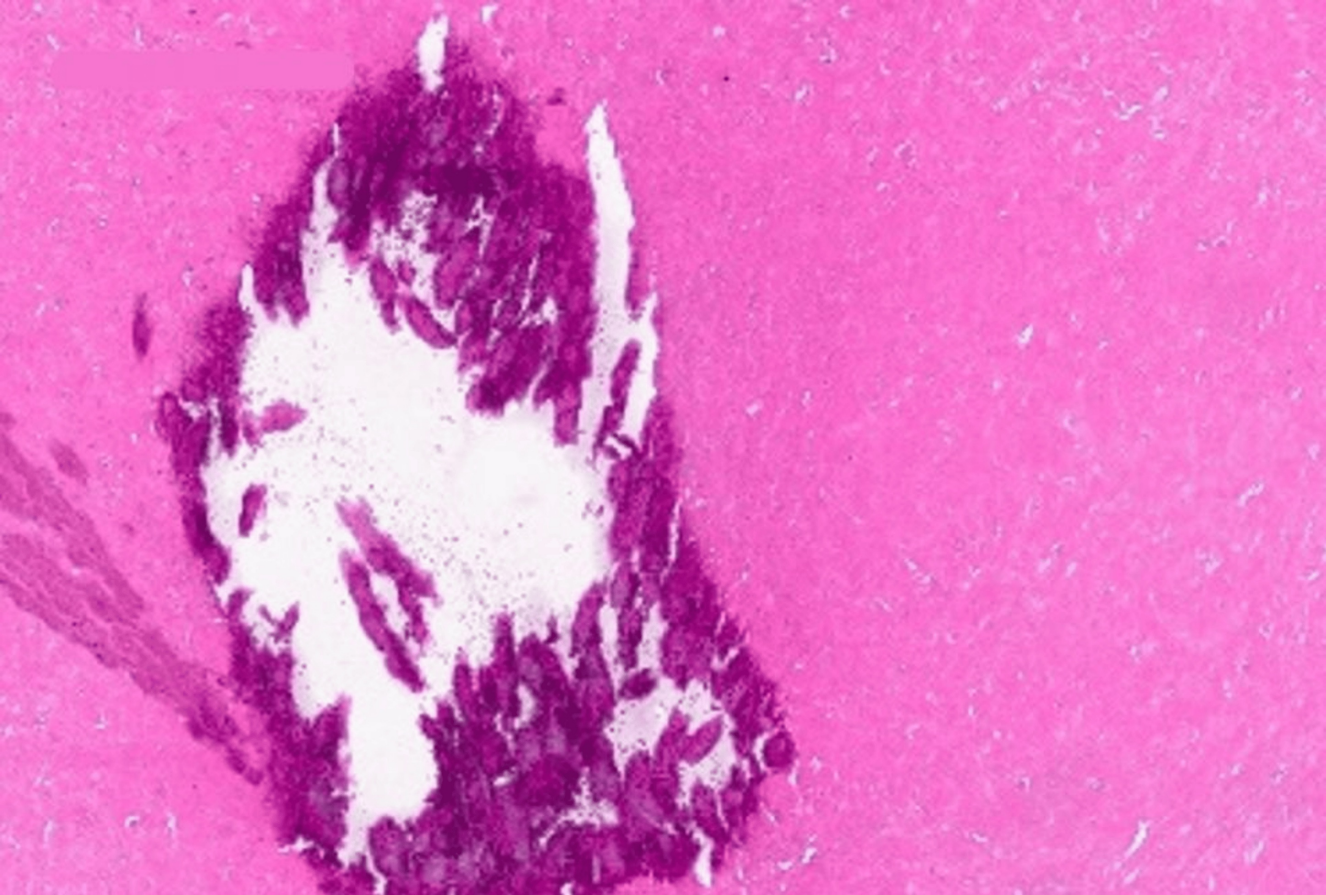
Cystic island (magnification ×20) illustrating calcification with hemorrhage

Histopathology analysis revealed a well-defined circumscribed silhouette with a lobular proliferation of squamous cystic islands filled with dense, thick keratin (Figure 4). The lining epithelium is a stratified squamous epithelium exhibiting abrupt trichilemmal keratinization (Figures 5, 6) with additional epithelial proliferation within the center of the cystic space (Figure 6). Also present are cholesterol clefts, calcification, and hemorrhage (Figure 7). Further, no mitotic activity marked cytologic atypia, perineurial or angiolymphatic invasion, or invasive growth was identified. Thus, a diagnosis of benign PPT was made.

Later, after one year in 2025, she presented for follow-up with enlargement of the other lesions that were initially smaller but had increased significantly over the last year until they became tender. The two main cysts that brought her to the clinic were two left parietal masses measuring 6 and 4.5 cm in diameter on recent CT scanning. The patient has been planned for future third surgical resection.

## Discussion

PPT or proliferating trichilemmal tumor (PTT) is a rare type of cutaneous tumors that predominantly affect elderly women. It was first described in the literature by Wilson Jones in 1966 in his study as a distinctive entity from squamous cell epitheliomas arising in cysts or as Malherbe's calcifying epithelioma [6]. The disease usually follows an autosomal dominant pattern, as shown in our case, since all her sisters, father, and grandmother suffer from the same condition, and the family is familiar with this condition. Hence, we called for other family members to undergo a full assessment [4]. No difference across all ethnic and racial backgrounds regarding disease incidence was found, and PTT affects all groups equally. However, it shows more preference for the female gender in the older age group [7].

The scalp is the most reported site, as more than 90% of the cases arise on the scalp, as shown in Table 1. Other reported possible sites are the back, vulva, nose, mons pubis, buttocks, wrist, chest, and elbow [8]. The tumor could present as a single or multiple masses simultaneously. As in our case, she presented for the first time with two lesions, and her recurrence was two other lesions.

**Table 1:** Selected scalp PPT cases in the last 25 years LN: lymph node; NA: not available; PPT: proliferating trichilemmal tumor

Study	Age (year)	Gender	Site	Size (millimeters)	Duration of growth	Treatment	Outcome	Follow-up period (months
Alarcón Pérez et al. [3]	59	F	Left parietal	18	6 years	Wide local excision	No recurrence	12
	29	F	Left parietal	NA	4 years	Mohs micrographic surgery	No recurrence	17
	71	M	Left occipital	50	3 years	Mohs micrographic surgery	No recurrence	10
Ye et al. [9]	41	M	Scalp (unspecified)	10	NA	Wide local excision	Recurrence after 3 months	3
	85	F	Scalp (unspecified)	90	NA	Wide local excision	Recurrence after 9 months	9
	75	F	Scalp (unspecified)	15	NA	Wide local excision	No recurrence	2
	44	F	Scalp (unspecified)	21	NA	Wide local excision	No recurrence	6
	78	F	Scalp (unspecified)	60	20 years	Wide local excision with LN dissection	No recurrence	12
	65	F	Scalp (unspecified)	30	1 year	Wide local excision	No recurrence	15
	44	F	Scalp (unspecified)	10	NA	Wide local excision	No recurrence	15
	57	F	Scalp (unspecified)	10	NA	Wide local excision	No recurrence	24
	66	F	Scalp (unspecified)	10	NA	Wide local excision	No recurrence	24
	87	M	Scalp (unspecified)	6	1 year	Wide local excision	No recurrence	54
	65	F	Scalp (unspecified)	30	NA	Wide local excision	No recurrence	102
	66	M	Scalp (unspecified)	23	3 months	Wide local excision	Lymph node metastasis	Died of disease after 5 months
	62	F	Scalp (unspecified)	25	1 month	Wide local excision	No recurrence	2
	86	F	Scalp (unspecified)	50	6 years	Wide local excision with LN dissection	No recurrence	36
Chang et al. [4]	69	F	Occipital and parietal	15	25 years	NA	NA	NA
Cavanagh et al. [7]	69	F	Right frontal	30	6 years	Wide local excision with adjuvant radiation therapy	No recurrence	NA
	53	F	Left frontal	15	10 years	Wide local excision	No recurrence	24
Alici et al. [10]	53	F	Scalp, unspecified (three masses)	5,3, 2.5	4 years	Wide local excision	No recurrence	NA
López-Ríos et al. [11]	97	F	Scalp (unspecified)	30	NA	Wide local excision	No recurrence	NA
Gulati et al. [12]	65	F	Occipital	25	9 years	Wide local excision	No recurrence	NA
Tikku et al. [13]	63	M	Left occipital	75	5 years	Wide local excision	No recurrence	NA
Erdem et al. [14]	70	F	Biparietal area (eight masses)	150 (the largest)	25 years	Wide local excision	NA	NA
Lobo et al. [15]	29	M	Left frontoparietal	300	7 years	Palliative radiotherapy with chemotherapy	Intracranial extension and lung metastasis	NA
Kemaloğlu et al. [16]	85	M	Anterior of the vertex	65	2 years	Wide local excision	Skull and dura invasion	7
	69	M	Behind the left ear	10	1 year	Wide local excision	Recurrence after 20 months with metastasis	Died from lung metastasis
	55	F	Right temporoparietal	90	1.5 years	Wide local excision	No recurrence	10
Mathis et al. [17]	51	F	Right occiput	10	6 years	Wide local excision	NA	NA
Morgado et al. [18]	61	M	Right and left frontoparietal	150	15 years	Surgical debridement	NA	NA
Stewart et al. [19]	77	F	Right parietal	50	10 years	Mohs technique	No recurrence	7

The primary characteristic of a PTT is a slow-growing scalp mass, typically large in size (2-15 cm), which is mobile and may be solid or partially cystic. These masses are usually found within the dermis or subcutaneous tissue of the scalp and are generally nontender. The masses can become exophytic and occasionally with possible ulceration. Similarly, our case mentioned an ulceration in one of the lesions, especially with increased size [15]. Although PTTs are generally benign, some cases in the literature reported an SCC development from previously existing PTT [20].

Similarly, some types of PTT tend to metastasize to regional lymph nodes or more distally. One study found a local metastasis rate of 3.7%-6.6% and a rate of 1.2%-2.6% for regional lymph node spread [21]. Further clinical sequelae include superimposed infection and pressure necrosis of adjacent tissues secondary to the growth of the cystic masses [21]. Various conditions resemble PTT on a clinical basis, for example, SCC, multiple skin nodules such as trichilemmoma, trichilemmal keratosis, clear-cell syringoma, hidradenoma, malignant hidradenoma, and sebaceous carcinoma [9].

PTT poses a challenge in diagnosis and is frequently misdiagnosed due to the scarcity of the disease as well as the shared histopathological features, such as cellular atypia, that are often misleading to SCC. However, the characteristic histological finding is the trichilemmal keratinization, which involves sudden, compact, amorphous keratinization of the epithelial cells that cover the cyst wall without a granular layer. Also, malignant transformation features noticed are cytologic pleomorphism, atypical mitoses, and infiltrative growth patterns [22]. Moreover, TC formation, trichilemmal type keratinization, eosinophilic hyaline membrane, calcification, and absence of premalignant epidermal lesion are features favoring the diagnosis of PTT rather than SCC [22].

Radiology has a limited role in disease diagnoses, but is important to assess the extent of the lesion, such as local invasion and distal spread. In cases where skull invasion is suspected, contrast-enhanced CT of the head is the modality of choice for the assessment of the bony compartment. Additionally, CT of the neck should be considered to evaluate regional and lymph node metastasis at the base of the skull and in the neck. Advanced imaging by magnetic resonance imaging is helpful for detecting dural involvement or other soft tissue invasion, which favors the diagnosis of malignant PTT [3].

Ye et al. studied over 70 cases of PTT and proposed a classification system that divides PTT into three groups according to clinical and histopathological features. The first group is considered benign since it shows no local or distal invasion with minimal or no nuclear atypia [9]. Moreover, immunohistochemistry stains strongly for CK10 and involucrin in this group. Group 2 or locally malignant PTT (LMPPT) presents as an irregular, locally invasive mass with extension to the deep dermis and subcutis with modest cytologic abnormalities [9]. Group 3 is characterized by high-grade malignant PPTs (HMPPTs) that are locally and distally invasive, with or without metastasis to other organs. These tumors are cytologically anaplastic, displaying significant nuclear atypia and pathological mitotic forms [9].

Concerning the prognosis, benign PPT surgical excision is considered curative, whereas the more aggressive management is needed for LMPPT and HMPPT, as wider surgical removal and additional therapeutic interventions [21]. Nevertheless, the rates of local recurrence found in the meta-analysis were 3.7% [9]. More recent reviews suggest that adjuvant radiotherapy, especially in the treatment of malignant proliferating trichilemmal tumors, further reduces the recurrence [22].

Unfortunately, to date, there is no standardized guideline regarding optimal management. However, surgical excision with lateral margins of at least 1 cm is usually considered for benign types, with alternative methods arising as Mohs surgery that shows promising results [23]. More complex management is required in the malignant types, such as lymph node dissection with adjuvant chemotherapy like cisplatin and 5-fluorouracil [24].

Radiotherapy appears to be a significant option either as isolated or adjuvant, especially in the elderly with tumors in cosmetically or functionally sensitive areas. Whereas, in the case of metastasis, palliative radiotherapy is added to the management [21]. Table 1 summarizes multiple selected scalp PTTs and their management with the follow-up results [3-24].

## Conclusions

PTT is a rare tumor of the hair-bearing area, most commonly in the scalp of elderly women. It is classified depending on the clinical and microscopical picture into different types. However, to date there are no clear consensus for treatment options. In our article, we describe for the first time in the literature a case of recurrent benign PTT reported in Saudi population that occurs in a younger age group compared to existing literature. Our treatment approach relies on surgical excision with multiple recurrence episodes. More cases need to be analyzed to generate standardized management guidelines for PTT disease.
